# Detection of ischemic penumbra using combined perfusion and T2* oxygen challenge imaging

**DOI:** 10.1111/ijs.12327

**Published:** 2014-07-15

**Authors:** Craig A Robertson, Christopher McCabe, M Rosario Lopez-Gonzalez, Graeme A Deuchar, Krishna Dani, William M Holmes, Keith W Muir, Celestine Santosh, I Mhairi Macrae

**Affiliations:** 1 Centre for Stroke and Brain Imaging Research, Institute of Neuroscience and Psychology, College of Medicine, Veterinary & Life Sciences, University of GlasgowGlasgow, UK; 2 Institute of Neurological Sciences, Southern General HospitalGlasgow, UK

**Keywords:** ischemic penumbra, neuroimaging, oxygen challenge, T2* MRI

## Abstract

**Background:**

Acute ischemic stroke is common and disabling, but there remains a paucity of acute treatment options and available treatment (thrombolysis) is underutilized. Advanced brain imaging, designed to identify viable hypoperfused tissue (penumbra), could target treatment to a wider population. Existing magnetic resonance imaging and computed tomography-based technologies are not widely used pending validation in ongoing clinical trials. T2* oxygen challenge magnetic resonance imaging, by providing a more direct readout of tissue viability, has the potential to identify more patients likely to benefit from thrombolysis – irrespective of time from stroke onset – and patients within and beyond the 4·5 h thrombolysis treatment window who are unlikely to benefit and are at an increased risk of hemorrhage.

**Aims:**

This study employs serial multimodal imaging and voxel-based analysis to develop optimal data processing for T2* oxygen challenge penumbra assessment. Tissue in the ischemic hemisphere is compartmentalized into penumbra, ischemic core, or normal using T2* oxygen challenge (single threshold) or T2* oxygen challenge plus cerebral blood flow (dual threshold) data. Penumbra defined by perfusion imaging/apparent diffusion coefficient mismatch (dual threshold) is included for comparison.

**Methods:**

Permanent middle cerebral artery occlusion was induced in male Sprague-Dawley rats (*n* = 6) prior to serial multimodal imaging: T2* oxygen challenge, diffusion-weighted and perfusion imaging (cerebral blood flow using arterial spin labeling).

**Results:**

Across the different methods evaluated, T2* oxygen challenge combined with perfusion imaging most closely predicted 24 h infarct volume. Penumbra volume declined from one to four-hours post-stroke: mean ± SD, 77 ± 44 to 49 ± 37 mm^3^ (single T2* oxygen challenge-based threshold); 55 ± 41 to 37 ± 12 mm^3^ (dual T2* oxygen challenge/cerebral blood flow); 84 ± 64 to 42 ± 18 mm^3^ (dual cerebral blood flow/apparent diffusion coefficient), as ischemic core grew: 155 ± 37 to 211 ± 36 mm^3^ (single apparent diffusion coefficient threshold); 178 ± 56 to 205 ± 33 mm^3^ (dual T2* oxygen challenge/cerebral blood flow); 139 ± 30 to 168 ± 38 mm^3^ (dual cerebral blood flow/apparent diffusion coefficient). There was evidence of further lesion growth beyond four-hours (T2-defined edema-corrected infarct, 231 ± 19 mm^3^).

**Conclusions:**

In conclusion, T2* oxygen challenge combined with perfusion imaging has advantages over alternative magnetic resonance imaging techniques for penumbra detection by providing serial assessment of available penumbra based on tissue viability.

## Introduction

Following an acute ischemic stroke, the ischemic penumbra represents electrically inactive and metabolically compromised but potentially viable tissue which is the target for acute stroke therapies. It ultimately becomes incorporated into final infarct unless salvaged by reperfusion. The thrombolytic agent alteplase (recombinant tissue plasminogen activator, rt-PA) is the only drug currently licensed to treat stroke. It may be administered within 4·5 h of stroke onset based on clinical symptoms and a noncontrast computed tomography (CT) scan to exclude intracerebral hemorrhage or major established hypodensity 1. Alteplase is designed to induce reperfusion by lysing occluding blood clots and significantly increases independent recovery, but carries a risk of brain hemorrhage (∼2%, increasing with delay in administration) and is underutilized. A UK report of thrombolysis rates reported that an overall 1·4% of acute stroke patients were thrombolysed, despite 14% of patients being eligible for thrombolysis 2.

Thrombolysis decisions may soon be informed by more advanced imaging, identifying a potential responder population based on individual pathophysiology. Magnetic resonance imaging (MRI) perfusion imaging (PI)/diffusion-weighted imaging (DWI) mismatch is widely accepted as a marker of penumbral tissue, while DWI/FLAIR mismatch may have utility in predicting lesion age and is under investigation as a selection marker for thrombolysis when stroke onset time is unknown 3,4. Clinical adoption of perfusion/diffusion mismatch is currently limited by the lack of standardized perfusion and diffusion thresholds, uncertain ability to differentiate penumbra from benign oligemia, and uncertainty whether DWI hyperintensity reflects irreversible damage, particularly at early time-points. Clinical trials have to date failed to support the utility of perfusion–diffusion mismatch in selection for thrombolysis 5,6. Alternative MRI techniques for penumbra detection are therefore worthy of investigation, particularly methods that may offer an index of tissue viability that is not dependent on interpretation of PI thresholds alone.

**Figure 1:**
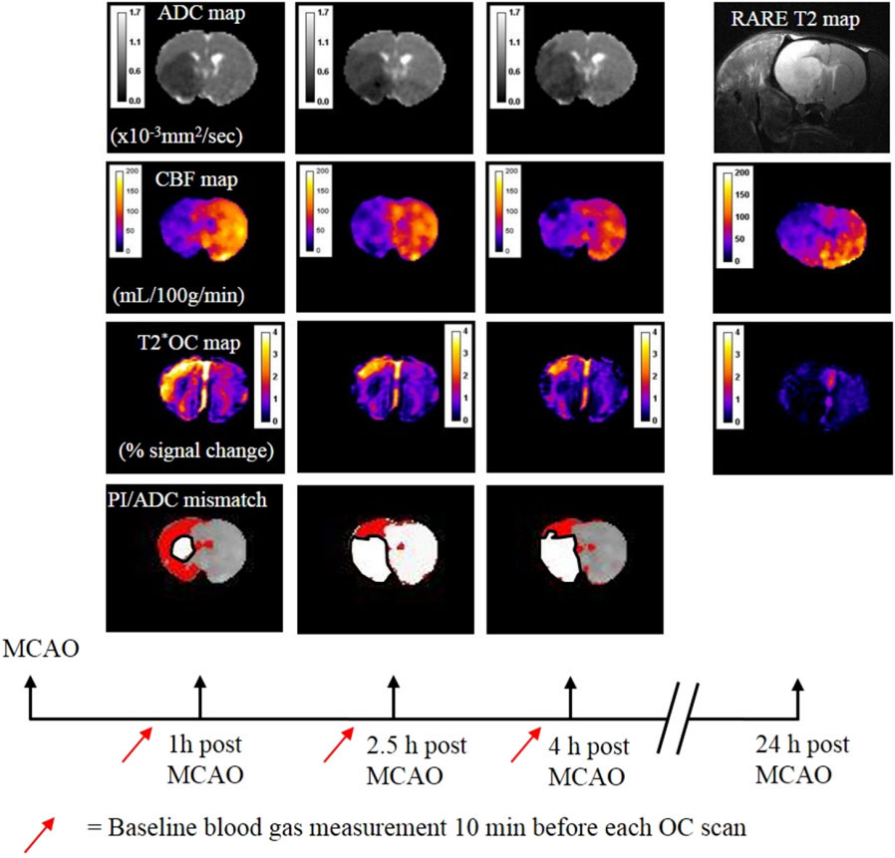
Multimodal MRI for one coronal slice in a representative rat and timeline of experimental protocol. Scanning commenced at approximately 1, 2·5, 4 and 24 h post-stroke: DWI to generate ADC maps, arterial spin labeling (ASL) to generate fully quantitative blood flow maps and mismatch images, a T2*OC scan to derive T2* percentage signal change maps, and a RARE T2 map to identify 24 h infarct. Red arrows on the timeline depict blood gas measurement 10 min before each OC scan. ADC, apparent diffusion coefficient; CBF, cerebral blood flow; DWI, diffusion weighted imaging; OC, oxygen challenge; MCAO, middle cerebral artery occlusion; MRI, magnetic resonance imaging; PI, perfusion imaging.

We have developed such an alternative approach for penumbra detection. T2* oxygen challenge (T2*OC) detects compensatory mechanisms in response to reduced cerebral blood flow (CBF), which may identify a distinct MRI tissue signature of penumbra. Penumbra displays a higher venous deoxyhemoglobin concentration than surrounding tissue due to increased oxygen extraction fraction (OEF) 7 and cerebral blood volume (CBV) 8. T2* MRI is sensitive to OEF and CBV changes as venous deoxyhemoglobin provides the contrast for blood oxygen level-dependent (BOLD) T2* imaging 9. T2*OC uses a transient hyperoxic challenge to identify different tissue compartments in the ischemic hemisphere, based on changes in deoxyhemoglobin : oxyhemoglobin ratio 10. Deoxyhemoglobin and free oxygen dissolved in plasma are paramagnetic and will reduce T2* signal, while diamagnetic oxyhemoglobin has minimal influence on T2*. Increased oxygen delivery during an OC converts deoxyhemoglobin to oxyhemoglobin, with a resultant increase in T2*-weighted signal which can be mapped as a percentage signal change (Fig. 1). Penumbra (and large veins) therefore displays the greatest increase in T2*-weighted signal during an OC, ischemic core displays the smallest change, and nonischemic tissue displays a small increase in signal.

T2*OC may provide insight into the dynamic behavior of hypoperfused tissue, and better prediction of tissue viability compared with the PI/DWI paradigm. A small proof of concept study in acute stroke patients 11 supports its potential clinical utility, and subsequent preclinical developments have improved technique sensitivity and ability to translate to the clinic 12.

## Aims

Compartmentalizing penumbra using two parameters (dual threshold analysis combining T2*OC with CBF values) rather than using a single T2* threshold may improve the technique further and provide a more accurate assessment of penumbra. We sought to compare single- and dual-threshold analysis for penumbra quantification using T2*OC and to evaluate its utility for serial scanning to track penumbra fate over the first four-hours in a rat permanent middle cerebral artery occlusion (MCAO) model. Serial PI and DWI (zero to four-hours), and 24 h T2-weighted imaging provided information on ischemia, acute tissue injury, and infarct, respectively, and alternative assessments of penumbra from PI/apparent diffusion coefficient (ADC) mismatch and ADC lesion growth.

## Methods

All experiments were performed under a UK Home Office license, were subject to the Animals (Scientific Procedures) Act (1986) and approved by the local University Ethical Review Panel. Figure 1 displays the full experimental protocol.

### Rodent MCAO surgery

Anesthesia was induced in male Sprague-Dawley rats (*n* = 6, 290–350 g, Harlan, Bicester, UK) with 5% isoflurane and maintained at 2% in medical air, enriched with oxygen (30%).

Body temperature was maintained at 37°C ± 0·5°C and a femoral artery cannulated for continuous blood pressure monitoring (AcqKnowledge, Biopac Systems, Goleta, CA, USA) and blood gas analysis. Permanent MCAO (pMCAO) was achieved with a 5-0 silicon rubber-coated intraluminal monofilament advanced to block the origin of the middle cerebral artery 13.

### MRI scanning

MRI data were acquired on a Bruker Biospec (Wikingerstrasse, Karlsruhe, Germany) 7-T/30-cm system equipped with an inserted gradient coil (121 mm ID, 400 mT/m) and a 72-mm birdcage resonator.

#### T2*-weighted imaging

A single-shot, gradient echo EPI sequence (TE: 20 ms, TR: 10 s, matrix 96 × 96, FOV 25 × 25 mm, 8 contiguous slices of 1·5 mm thickness, 2 averages, temporal resolution 20 s, 30 repetitions) was used to assess T2*-weighted signal change during OC. The paradigm for the T2*-weighted OC sequence was five-minutes breathing air, followed by five-minutes breathing 100% oxygen, and then 10 mins breathing air. Four coronal MRI slices throughout MCA territory, selected to minimize the effect of susceptibility distortion around the ear canals, were used to generate T2*-weighted signal change maps using ImageJ (http://rsb.info.nih.gov/ij/). The average baseline signal (over five-minutes) was subtracted from the peak signal during OC, then divided by the average baseline signal and multiplied by 100.

#### DWI and PI scanning

DWI [spin-echo planar (SE-EPI) TE: 43 ms, TR: 4000·3 ms, matrix 96 × 96, FOV 25 × 25 mm^2^, 3 directions: x, y, z, B values: 0, 1000 s/mm^2^, 8 slices of 1·5 mm thickness] was performed to assess ischemically injured tissue and it was possible to collect data from eight slices, four of which anatomically matched PI and T2*. Noninvasive quantitative CBF was carried out to identify the perfusion deficit on four 1·5-mm thick coronal slices throughout the MCA territory using a form of pseudo-continuous arterial spin labeling based on a train of adiabatic inversion pulses 14,15. The sequence employs a spin-echo EPI imaging module (TE 20 ms, TR 7000 ms, matrix 96 × 96, FOV 25 × 25 mm, slice thickness 1·5 mm, 16 averages, 4 shots) preceded by 50 hyperbolic secant inversion pulses in a 3s train.

#### T2-weighted imaging

A coronal RARE T2 sequence (effective TE: 46·8 ms, TR: 5000 s; in plane resolution 97 μm; 30 slices, 0·75 mm thickness) was used for T2-derived infarct measurement at 24 h post-stroke.

### MRI data analysis for penumbra assessment

Volumes of tissue with abnormal T2*OC response were determined using two approaches: (1) applying a single threshold [mean plus 2 standard deviations (SDs) of the contralateral hemisphere, excluding the ventricles, venous sinuses, and large veins] to T2*OC maps in ImageJ to derive tissue volumes 8,16; (2) voxel-based analysis using T2* and CBF data and thresholds to categorize tissue into four compartments (T2*OC-defined penumbra, ischemic core, normal tissue and veins/venous sinuses, see Fig. 3a). PI and DWI data were analyzed in two ways, (1) voxel-based analysis using ADC and CBF thresholds to categorize tissue into five compartments: PI/ADC mismatch, ischemic core, normal, negative mismatch, and ventricles; and (ii) penumbra volume derived from ADC lesion growth using an in-house absolute ADC threshold.

Quantitative ADC maps (in mm^2^/s) were calculated using Paravision (Bruker BioSpin, Ettlingen, Germany) and subsequently analyzed using ImageJ. CBF maps were also generated using ImageJ. The ADC lesion was thresholded using an in-house absolute ADC value of 0·59 × 10^−3^ mm^2^/s, which when applied to data at four-hours post-stroke generated an ADC lesion which matched 24 h (brain swelling corrected) infarct following permanent MCAO in Sprague-Dawley rats.

#### Coregistration

T2* images were linearly coregistered to corresponding ADC slice maps using Analyze (AnalyzeDirect, Inc., Overland Park, KS, USA). Following coregistration, T2*, ADC and CBF thresholds were applied to relevant images (four coronal slices within MCA territory) for voxel-based analysis and volumetric measurements of tissue compartments.

#### Voxel-based analysis

Voxel-based analysis, generated using codes written in Matlab (MathWorks, Natick, MA, USA) allowed unbiased analysis of the spatial, volumetric, and dynamic evolution of penumbra and ischemic core. Tissue was compartmentalized using thresholds set for two parameters: (1) T2* signal change and CBF for T2*OC data sets; and (2) ADC and CBF data sets for PI/ADC mismatch. Color-coded images were derived based on T2*OC signal change and CBF values, or ADC and CBF values enabling analysis of temporal changes in voxel distribution across ischemic core, penumbra, and nonischemic tissue.

The number of voxels within each tissue compartment was counted and converted to an area by multiplying the number of pixels by the pixel area (0·0678 mm^2^) and then a volume by multiplying the areas by slice thickness (1·5 mm) and adding the volumes for the four consecutive slices. Group data were generated for each tissue compartment at each acute time-point poststroke for T2*and CBF, ADC/CBF, and from the single T2* threshold data sets.

Large veins and venous sinuses were segregated from the main tissue compartments by excluding tissue with elevated T2* (>mean + 2 SD of contralateral hemisphere) and CBF (values above threshold – 57% reduction of mean contralateral CBF). Ventricles were excluded from the ADC/CBF data sets by applying an ADC threshold value (0·96 × 10^−3^ mm^2^/sec).

For T2*OC/CBF analysis, the four compartments were:

- T2*OC-defined penumbra: T2* signal change above set threshold, CBF value below set threshold
- *Ischemic core*: T2* signal change below set threshold, CBF below set threshold
- *Normal*: T2* signal change below and CBF above set thresholds, and
- *Veins and venous sinuses*: both T2* signal change and CBF above set thresholds

For ADC/CBF analysis, the five compartments were:

- *PI/ADC mismatch*: ADC value above set threshold, CBF value below set threshold
- *Ischemic core*: both ADC and CBF values below set thresholds
- *Normal*: both ADC and CBF values above set thresholds
- *Negative mismatch*: ADC value below threshold, CBF value above threshold, and
- *Ventricles*: Above an ADC threshold of 0·96 × 10^−3^ mm^2^/s

#### ADC lesion expansion

ADC lesion expansion from one to four-hours was subtracted from 24 h infarct data to provide an alternative assessment of penumbra.

#### ADC lesion expansion

The absolute ADC threshold of 0·59 × 10^−3^ mm^2^/s was applied, and the volume of the ADC lesion quantified by multiplying the area on each slice by 1·5 (slice thickness) and summing the data for either four or eight slices. Penumbral volume was derived by subtracting the ADC lesion volume at each time-point from the swelling-corrected 24 h infarct volume and was quantified over four slices, for direct comparison with T2*OC and PI/ADC mismatch and for all eight slices available from DWI.

#### Quantifying infarct volume at 24 h

Infarct was calculated from regions of hyperintensity on the RARE T2 scans and corrected for ipsilateral brain swelling and contralateral compression 17. Eight coronal RARE T2 slices which matched the four-hour acute ADC slices were selected, and the ipsilateral and contralateral hemisphere areas, as well as 24 h infarct areas, were calculated by manual tracing using ImageJ. The volumes of the ipsilateral and contralateral hemispheres and the infarct were calculated by summing the individual areas and multiplying by slice thickness (0·75 mm).

#### Prediction error

Ischemic core and penumbra volumes were summed and subtracted from edema-corrected infarct to calculate a prediction error for each method.

### Statistical analysis

Data are presented as mean ± SD. Comparison of physiological variables, lesion evolution, and volumetric differences in ischemic core and penumbra across the four methods were analyzed using one-way ANOVA with Student's paired *t*-test and Bonferroni correction. A paired *t*-test was used to analyze ADC-derived lesion volume at four-hours and T2-defined infarct at 24 h.

## Results

### Physiological variables

Physiological variables [apart from partial pressure of oxygen in arterial blood (PaO_2_) during OCs] were maintained within physiological limits. Recordings collected immediately before each OC (Table 1) displayed no significant differences in partial pressure of carbon dioxide in arterial blood (PaCO_2_) or PaO_2_. A small increase in blood pH was recorded at four-hours compared with one-hour and 2·5 h time-points (*P* < 0·0001).

**Table 1:** Physiological variables

	One-hour scan	2·5 h scan	Four-hour scan
pH	7·34 ± 0·04	7·34 ± 0·05	7·36 ± 0·04*
PaCO_2_	44·5 ± 5·7	45·3 ± 10	42·4 ± 5·7
PaO_2_	93·6 ± 11	93·4 ± 12	95·7 ± 15

* *P* < 0·0001, one-way ANOVA followed by Student's paired *t*-test with a Bonferroni correction.

Physiological variables. Data from arterial blood samples collected immediately before each OC. Data expressed as mean ± SD, *n* = 6.

### T2*OC -defined penumbra

#### Single threshold analysis

**Figure 2:**
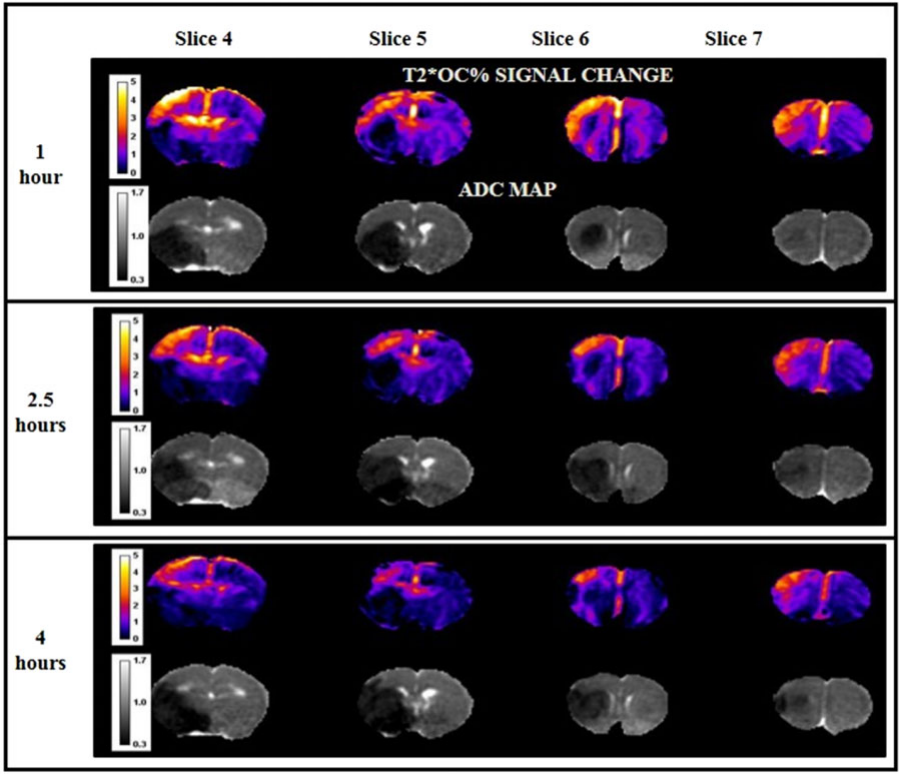
T2*OC percentage signal change maps and equivalent ADC maps over four coronal slices at 1, 2·5, and 4 h post-stroke in a representative animal. T2*OC maps show a reduction in penumbral tissue from one to four-hours post-stroke, and ADC maps display a concomitant increase in ADC lesion. Calibration bar on T2* maps, in units of percentage signal change and on ADC maps, in ×10^−3^ mm^2^/s. ADC, apparent diffusion coefficient; OC, oxygen challenge.

During OC, an increased T2* percentage signal change, compared with surrounding and contralateral tissue, was apparent in dorsolateral cortex (Fig. 1). This increased signal lay within the perfusion deficit adjacent to the diffusion abnormality (Figs 1 and 2). The mean volume of tissue defined as penumbra was 77 ± 44 mm^3^ at one-hour post-stroke and gradually declined over subsequent time-points (Table 2) as the volume of ADC-defined core increased. OC repeated at 24 h revealed no increased T2* signal in tissue previously identified as penumbra (Fig. 1, Table 2).

**Figure 3:**
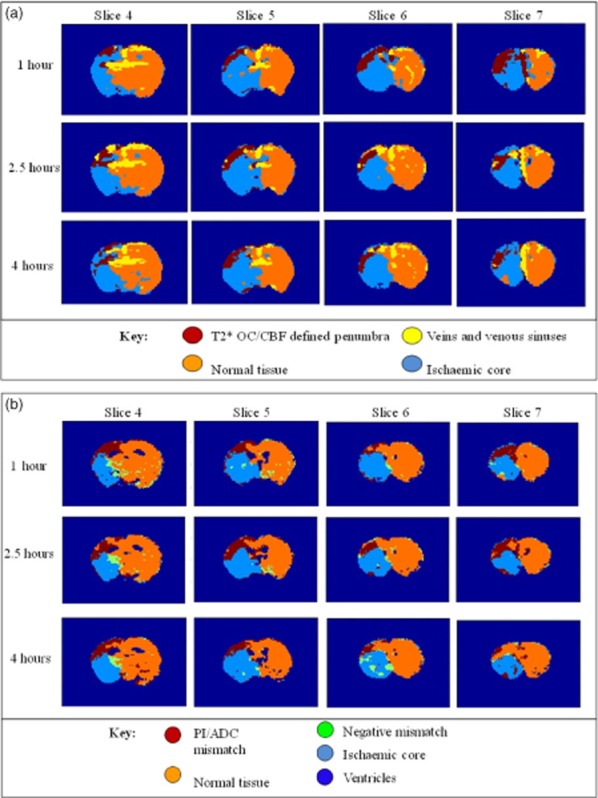
(a) Representative color-coded maps of T2*OC/CBF over four slices and three time-points post-stroke. Voxel-based analysis was performed by applying the CBF (57% reduction in mean contralateral values) and T2* (mean + 2 SD) thresholds. There was an expansion in ischemic core with concomitant loss of penumbral from one to four-hours post-stroke. Note that veins and venous sinuses (yellow shading) are differentiated from penumbral tissue, due to high T2* value and CBF above thresholds. (b) Equivalent color-coded maps of PI/ADC mismatch in the same animal. Voxel-based analysis performed using the same CBF threshold and an absolute ADC threshold of 0·6 × 10^−3^ mm^2^/s. ADC, apparent diffusion coefficient; CBF, cerebral blood flow; OC, oxygen challenge; PI, perfusion imaging.

#### Dual threshold analysis

Combining T2*% signal change and CBF data, voxels defining penumbra were clustered in the dorsolateral cortex, with the remaining voxels categorized as ischemic core, normal, or venous sinuses/veins (Fig. 3a). Volumetric data for penumbra and ischemic core at three time-points are shown in Table 2. Normal and venous compartments remained stable throughout (data not shown).

**Table 2:** Volumetric assessment of ischemic penumbra and ischemic core

	one-hour post-stroke	2·5 h post-stroke	Four-hours post-stroke	24 h post-stroke
Penumbra (mm^3^, *n* = 6)				
Using *single* (T2^*^) threshold	77 ± 44	56 ± 38	49 ± 37	0
	(range: 34–140)	(range: 15–110)	(range: 12–114)	
Using *dual* (T2^*^/CBF) thresholds	55 ± 41	39 ± 22	37 ± 12	0
	(range: 14–131)	(range: 4–76)	(range: 3–73)	
PI/ADC mismatch	84 ± 64	52 ± 41	42 ± 18	0
Using *dual* (CBF/ADC) thresholds	(range: 40–209)	(range: 19–129)	(range: 18–72)	
Derived from ADC lesion growth	78 ± 37	43 ± 34	26 ± 29	0
	(range: 35–117)	(range: 0·41–84)	(range: 13–83)	
Ischemic core (mm^3^, *n* = 6)				
Using *single* (ADC) threshold	155 ± 37	195 ± 44	211 ± 36	N/A
	(range: 99–201)	(range: 134–227)	(range: 164–251)	
Using *dual* (T2^*^/CBF) thresholds	178 ± 30	184 ± 41	205 ± 33	N/A
	(range: 109–211)	(range: 143–218)	(range: 162–248)	
Using *dual* (CBF/ADC) thresholds	139 ± 30	155 ± 32	168 ± 38	N/A
	(range: 96–168)	(range: 118–199)	(range: 119–212)	
24-h infarct, corrected for brain swelling (*n* = 5)	N/A	N/A	N/A	231 ± 19 (range: 205–249)

Volumetric assessment (calculated over four coronal slices) of ischemic penumbra using the single T2^*^ threshold and dual (T2^*^/CBF and CBF/ADC) thresholds; ischemic core using the single ADC threshold, and dual (T2^*^/CBF and CBF/ADC) thresholds; 24-h edema-corrected infarct. One-way ANOVA followed by Student's paired *t*-test with Bonferroni correction. There was no statistically significant difference between penumbral volumes assessed by the four different methods at any time-point. ADC, apparent diffusion coefficient; CBF, cerebral blood flow; PI, perfusion imaging.

### PI/ADC mismatch-defined penumbra

#### Dual threshold voxel-based analysis

Equivalent ADC/CBF maps revealed a similar pattern of penumbra, ischemic core, and normal voxels (Fig. 3b). The fourth compartment (CBF above threshold, ADC below threshold) defined a small number of negative mismatch voxels. Volumetric data for penumbra and ischemic core are shown in Table 2. Normal and negative mismatch compartments remained stable throughout (data not shown).

### ADC lesion growth-defined penumbra

Penumbra defined by subtracting the initial ADC lesion (over the same four slices) from the 24-h T2-defined (edema-corrected) infarct is presented in Table 2 for comparison. ADC data were also available across a greater rostro-caudal range (eight coronal slices). Penumbral volume determined from ADC lesion growth across eight slices was 168 ± 59 mm^3^ at one-hour, 119 ± 50 mm^3^ at 2·5 h, and 92 ± 48 mm^3^ at four-hours post-stroke.

### Comparison of methods for penumbra and ischemic core detection

Volumetric (Table 2) and spatial differences (Fig. 3) in tissue compartments were apparent between T2*OC/CBF and PI/ADC mismatch-defined penumbra, although differences across the four methods were not statistically significant. At one-hour poststroke, for penumbra volume determined from T2*OC/CBF, 69% of voxels were also determined to be penumbra on PI/ADC mismatch, and for penumbra volume determined from PI/ADC mismatch, 44% of voxels were also determined to be penumbra on T2*OC/CBF. The equivalent figures were 59% and 55%, respectively, at 2·5 h, and 47% and 30%, respectively, at four-hours post-stroke.

**Figure 4:**
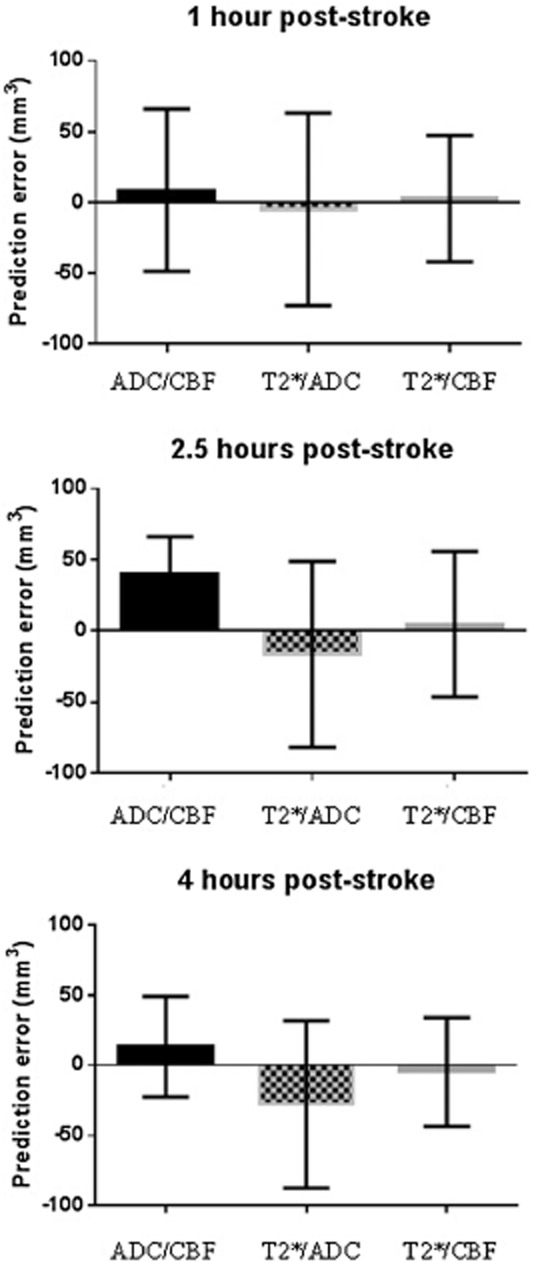
Prediction error for different methods of penumbra and ischemic core analysis. Penumbra and ischemic core volumes, calculated using each of the three methods (dual ADC/CBF, single T2*OC + ADC lesion, and dual T2*OC/CBF), were added together and subtracted from 24 h (edema-corrected) infarct volume to generate prediction errors (mean ± SD, *n* = 5). ADC, apparent diffusion coefficient; CBF, cerebral blood flow; OC, oxygen challenge.

**Figure 5:**
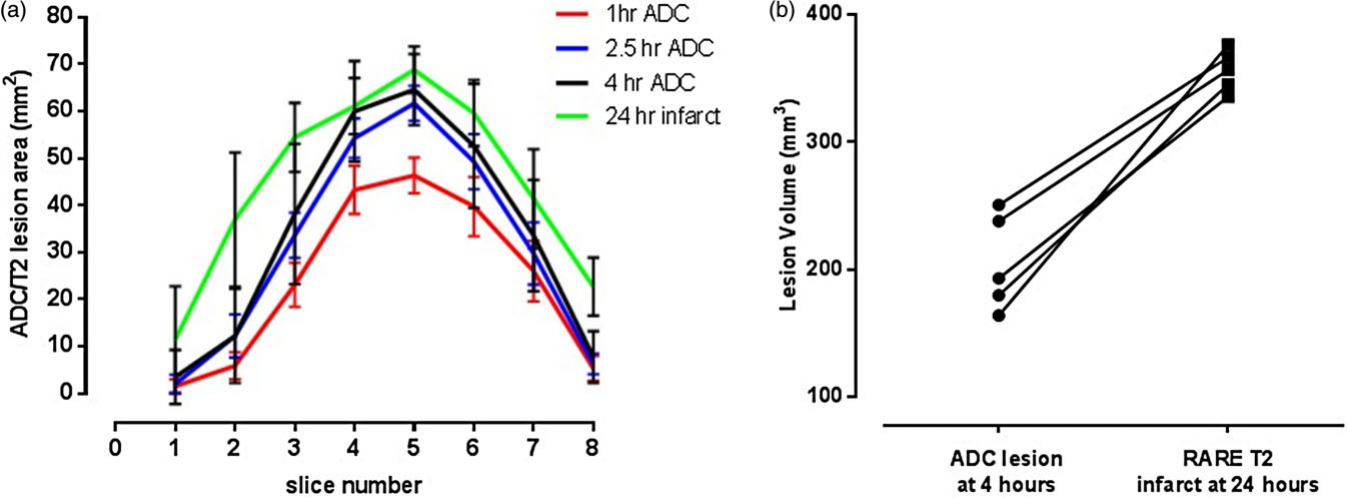
(a) ADC lesion areas at the three acute time-points and 24 h RARE T2-edema corrected infarct displayed across all eight forebrain slices and presented as mean ± SD. ADC lesion expansion (and concomitant mismatch loss) over the first four-hours was greatest within the four central coronal slices (3–6). Subsequent increases in ischemic damage were greatest in the caudal (1–3) and rostral (8) poles. ADC lesion volume increased significantly over time (*P* < 0·05, one-way ANOVA with Bonferroni correction). (b) Equivalent ADC-derived lesion volume and T2-derived infarct volume at 4 h and 24 h, respectively (*P* < 0·001, Student's paired *t*-test). The ADC lesion volume calculated over eight slices at 1, 2·5, and 4 h was 191 ± 51 mm^3^, 249 ± 51 mm^3^, and 356 ± 16 mm^3^, respectively (one to four-hours, *P* < 0·05). ADC, apparent diffusion coefficient.

**Figure 6:**
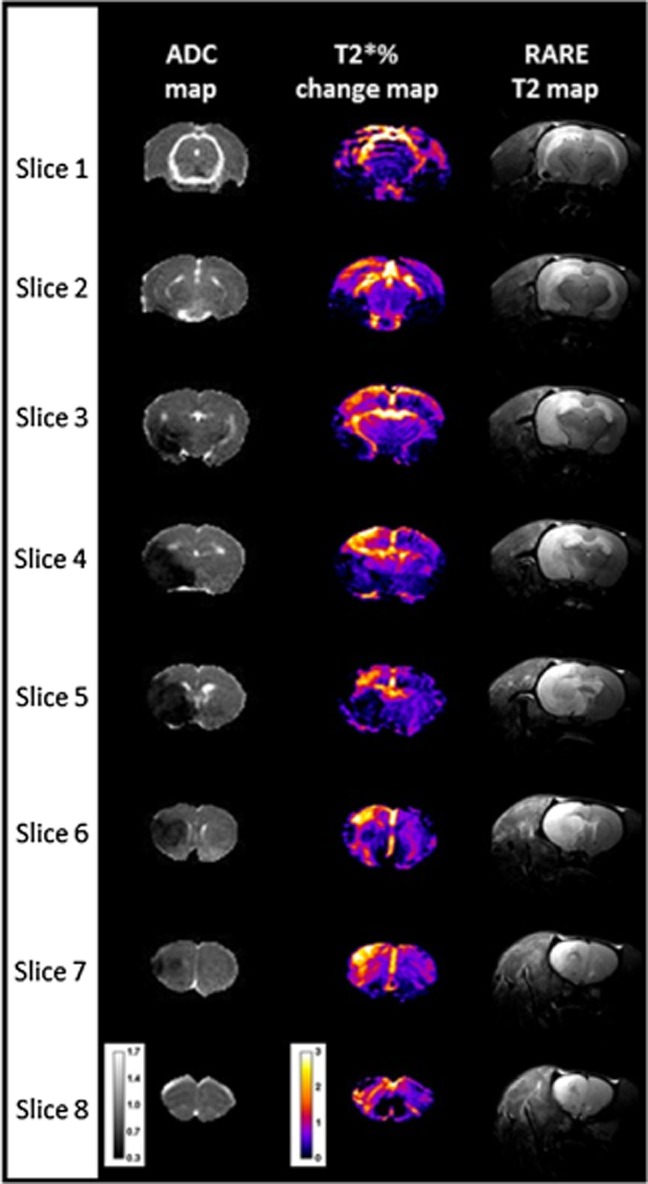
T2*OC-defined penumbra at four-hours post-stroke over eight coronal slices with accompanying ADC maps, and 24 h RARE T2 scans from a representative rat. Calibration bar on T2* maps, in units of percentage signal change and on ADC maps, in ×10^−3^ mm^2^/s. ADC, apparent diffusion coefficient; OC, oxygen challenge.

The sum of penumbra and ischemic core volumes, for the different methods, was compared with (edema-corrected) infarct volume to assess their predictive value (Fig. 4). Dual threshold T2*/CBF produced the smallest prediction error at all three time-points. All four methods provide evidence of remaining penumbra at the four-hour time-point. Four-hour ADC lesions compared with 24 h T2-defined infarct revealed evidence for further lesion growth beyond four-hours (Figs 5 and 6).

## Discussion

Positive identification of penumbra is not employed in the current imaging selection for intravenous rt-PA administration, which is based on exclusion of intracerebral hemorrhage or extensive established ischemic damage on noncontrast CT. While several observational clinical studies support perfusion/diffusion mismatch as a penumbral marker that may identify a ‘responder’ population, both within three-hours of onset and at times up to nine-hours after onset 18,19, a differential treatment effect in imaging-selected patients is not yet supported by randomized controlled trial data 6,20. PI/DWI mismatch is limited by the lack of standardized perfusion and diffusion thresholds 21,22 and small size of clinical data sets from which thresholds are derived. Assumptions that underlie the interpretation of DWI- and PW-abnormal tissue are questioned by findings including the reversibility of some DWI lesions with early reperfusion 23 and a heterogeneous metabolic state on concurrent positron emission tomography (PET) from voxels within acute DWI lesions 24. A fundamental limitation of current MR penumbral imaging is the inability to detect metabolic activity. By offering a dynamic measure of deoxyhemoglobin concentration that reflects variations in OEF (therefore, tissue metabolic capacity) and CBV, the T2*OC technique may better discriminate penumbra, healthy tissue, and ischemic core compared with existing MRI mismatch techniques.

This study provides evidence for the utility of T2*OC serial imaging in preclinical acute stroke research, and improved predictive value when T2*OC is combined with CBF data using voxel-based analysis. The maintenance of the increased T2* signal during OC and its return to baseline following OC is consistent with T2*-weighted signal change reflecting differences either in penumbral oxygen consumption or vasculature response to OC compared with surrounding tissue 10,11,25,26. Earlier validation studies with single time-point T2*OC also confirmed that the region of increased T2* signal exhibited characteristics of penumbra, that is, hypoperfused tissue with ongoing glucose metabolism 25 and recovery after early reperfusion 26.

As expected, a measureable volume of penumbra was detected with all four analysis methods at one-hour post-stroke and decreased in size with time, as tissue became incorporated into the ischemic core. Because a significant volume of penumbra was detected using all four methods at four-hour post-stroke, the OCs *per se* may have supported penumbra tissue and attenuated ADC-lesion growth during the scanning session. In previous studies using this rat strain and stroke model, where no OCs were applied, less than 10 mm^3^ of mismatch remained by three to four-hours post-stroke. There is evidence in the literature supporting the potential of oxygen to prevent penumbra loss. In a published study where PaO_2_ was maintained at 235–240 mmHg, no deterioration in mismatch volume was apparent over the first four-hours post-stroke 27. Studies investigating normobaric hyperoxia have reported 100% O_2_ attenuated ADC lesion growth without altering CBF, thereby preserving CBF/ADC mismatch, and reducing infarct volume in permanent and transient MCAO models 28–30.

### Limitations and further improvements

This pilot study was not designed to establish the superiority of one method over another, but to explore the relationship between T2*OC responsiveness and blood flow in the evolving stroke lesion: as such, it was not powered to detect statistical differences between techniques. The data demonstrate a trend to improved prediction of final infarct volume by T2*OC combined with perfusion imaging, which needs to be confirmed in a larger study. The potential for translation of T2*OC as a clinical imaging method has been established 11. A new clinical study is under way to optimize the T2*OC technique on scanners of different field strength, using healthy volunteers, a controlled human model system of brain hypoperfusion, and a homogeneous clinical population with acute stroke. Further refinements of the technique include strategies to increase the T2* signal to OC and reduce the % oxygen delivered 12 which will be translated to the clinic in the future.

Finally, improvements in penumbra detection may be possible with susceptibility-weighted imaging. These sequences measure magnitude information, but they also exploit phase information, thereby producing T2*-weighted sequences which are exquisitely sensitive to the dephasing effects of paramagnetic substances such as deoxyhemoglobin, and provide enhanced contrast over conventional gradient echo sequences.

## Summary

Dual threshold T2*OC/CBF voxel-based analysis identified tissue with characteristics of penumbra within anterior cerebral artery/MCA collateral supply territory, which decreased in size over time. When compared with alternative methods, T2*OC combined with CBF produced the smallest error in predicting subsequent infarct volume. The additional information it delivers, complementary to current MRI techniques for stroke diagnosis, supports its potential use within the routine stroke imaging protocol and adds to evidence promoting T2*OC as a valuable adjunct to PI/DWI imaging for penumbra detection. There was some evidence that repeat OCs delayed the evolution of stroke-induced damage, but more prolonged normobaric hyperoxia and strategies (e.g. intravenous oxygen carriers) to improve oxygen delivery to penumbra are required to influence final outcome.
